# Dr. Corydon J. Phillips

**Published:** 1901-03

**Authors:** 


Dr. Corydon J. Phillips, of Jamestown, N.Y., died at his home in that city, February 8, 1901, aged 60 years. He was an eclectic physician and a veteran of the civil war.

